# Food allergy management from the perspective of patients or caregivers, and allergists: a qualitative study

**DOI:** 10.1186/1710-1492-6-30

**Published:** 2010-11-30

**Authors:** Ya S Xu, Sam B Waserman, Susan Waserman, Lori Connors, Kristin Stawiarski, Monika Kastner

**Affiliations:** 1 Department of Pediatrics, McMaster University, Hamilton, Ontario, L8N 3Z5, Canada; 2 Research Associate, Centre for Health Economics and Policy Analysis, McMaster University, Hamilton, Ontario, L8N 3Z5, Canada; 3 Department of Medicine, McMaster University, Hamilton, Ontario, L8N 3Z5, Canada; 4 Bachelor of Health Sciences Program, Faculty of Health Sciences, McMaster University, Hamilton, Ontario, L8N 3Z5, Canada; 5 Department of Health Policy, Management and Evaluation, Faculty of Medicine, University of Toronto, Toronto, Ontario, M5T 3M6, Canada

## Abstract

**Background:**

Research has shown that the long term management of food allergy is suboptimal. Our study aims to provide direction for improvement, by evaluating food allergy management from the perspective of, food allergic patients or their caregivers, and allergists in selected outpatient settings in Ontario.

**Methods:**

This two-part study included an anonymous questionnaire completed by patients or their caregivers in allergy clinics, and a qualitative interview with allergists. In Part A, food allergic patients or their caregivers were surveyed about information they received on food allergy, their level of confidence with self-management, and their learning needs. In Part B, allergists were interviewed about teaching priorities and the challenges and strategies that currently exist in food allergy management. The questionnaire was developed and piloted at the Hamilton Health Sciences Corporation-McMaster University Medical Center Site. Using convenience sampling, participants were recruited from 6 allergy clinics in 5 Ontario cities. Patients of any age with food allergy who were evaluated by an allergist were considered for inclusion. Quantitative data was analyzed using descriptive statistics and frequency analysis. Audio recorded interviews with allergists were transcribed verbatim and analyzed using content analysis of grounded theory methodology.

**Results:**

Ninety-two food allergic families in the care of 6 allergists in Toronto, Hamilton, London, Kitchener, and Kingston participated in the study. Key areas requiring improvement in food allergy management were identified: 33% of families were not shown how to use an epinephrine auto-injector with a trainer, only 57% were asked to demonstrate an auto-injector, despite being on average at their 5th visit, and only about 30% felt very confident about when and how to give an auto-injector. Fifty percent of families did not receive sufficient information on medical identification and 21% did not receive information about support groups. Interviews with allergists revealed limitations in time and nursing resources.

**Conclusions:**

Our study highlights the educational gaps and overall experiences of food allergic families in Ontario, and the challenges faced by the allergists managing them.

## Background

Food allergy is an adverse immune response to food protein(s). Exposure to the culprit food(s) can result in a wide range of clinical responses, ranging from urticaria to anaphylaxis, the most severe form of allergic reaction. Food allergy affects about 5% of young children and 3% to 4% of adults in western countries [1]. Avoidance of food allergens and treatment of acute reactions with epinephrine are the mainstays of treatment [1].

Review of the literature shows that management of patients with food allergy is suboptimal. Studies on mortality in Ontario due to food related anaphylaxis showed that delayed or no administration of epinephrine was a factor in many of the deaths [2]. Similar trends were seen in US mortality data recorded by the American Academy of Allergy, Asthma & Immunology: between 1994 and 1999, only 3 of 32 individuals had epinephrine available for use at the time of their reaction [3]. A subsequent study found that of the 31 additional deaths between 2001 and 2006, only 4 individuals had epinephrine administered in a timely manner [4]. A recent systematic review investigating gaps in anaphylaxis management found 202 gaps. Deficiencies were demonstrated in knowledge and appropriate management of food allergy at multiple levels including physicians, patients, and their communities [5]. Practical strategies are needed to address these numerous gaps.

Our study aimed to provide direction for improvement in outpatient management by examining the experiences and educational needs of food allergic families, and the challenges faced by the allergists managing them.

## Methods

### Study design

This was a two-part study consisting of an anonymous self-administered questionnaire completed by patients with food allergy or their caregivers (Part A) and a qualitative interview of allergists (Part B). The questionnaire and interview questions were designed with input from allergists, a health research methodologist, and a patient support organization. The study was approved by the McMaster University Health Sciences Research Ethics Board.

#### Part A: The self-administered patient/caregiver questionnaire

The patient and caregiver questionnaires were piloted with 30 patients from the outpatient allergy clinic at McMaster University. Participants were asked to complete the questionnaire and to provide feedback on questions which they found unclear or difficult to understand. Data from the pilot assessment was used to ensure that the final questionnaire was clear, readable and complete (see Additional file 1).

During the study, informed consent or assent (where applicable), were obtained for all participants. For patients 15 years and younger, the caregiver answered the questionnaire on the patients' behalf. Patients age 16 and older were given the option to complete the questionnaire, depending on their level of maturity and interest. Patients of any age who were evaluated by an allergist were considered for inclusion. Patients who did not have food allergy or had a limited understanding of English were excluded from the study.

#### Part B: Semi-structured interview with allergists

Using convenience sampling, allergists who managed patients with food allergy across Ontario were recruited via email, from a list of allergists generated from the College of Physicians and Surgeons of Ontario website. Physicians who agreed to participate signed a consent form, completed a short demographics questionnaire, and then were interviewed by one of the researchers. The interviews were audio taped and transcribed verbatim for analysis.

### Data analysis

Quantitative data from the questionnaires (e.g. demographic and dichotomous data) were analyzed using descriptive statistics and frequency analysis. Qualitative questions from the questionnaire and transcribed physician interviews were analyzed using content analysis of grounded theory methodology [6,7]. In the event of missing data on the questionnaires, all questions and existing responses were considered for analysis.

## Results

### Demographic information (see Table 1 and Table 2)

**Table 1:** Characteristics of questionnaire participants (N =92)*

Characteristic	Mean (SD)
Patient age	8.8 (7.8)

Age of first allergic reaction	3.8 (6.9)

Mean number of visits to allergy clinic	4.6 (7.6)

Mean wait time (weeks) for clinic appointment	12 (15)

**Type of food allergy**	**N (%)**

Peanut	63 (68%)

Other nuts	49 (53%)

Egg	49 (53%)

Milk	18 (20%)

Seafood	16 (17%)

Sesame	7 (8%)

Soy	4 (4%)

Other food allergy	17 (18%)

*SD = standard deviation

**Table 2:** Characteristics of allergists (N = 6)

Characteristic		N (%)
**Age range (years)**	36-45	3 (50%)
	
	45-55	3 (50%)

**Gender**	Female	4 (67%)
	
	Male	2 (33%)

**Years in practice**	5-10	2 (33%)
	
	11-15	2 (33%)
	
	16-25	1 (17%)
	
	>25	1 (17%)

**Practice location**	Urban	6 (100%)

**Type of practice**	Academic	4 (67%)
	
	Community	2 (33%)

Questionnaire data was collected from 92 food allergic patients (mean age 8.8 years) or from their caregivers from Ontario. Fifteen of the participants were patients (16% of total) and 77 were caregivers (84% of total). Patients had visited their allergist on average 4.6 times. Six allergists were interviewed (age range 36-55 years) and the majority had been in practice 5-15 years (67%). They worked in Toronto, Kitchener, Hamilton, London and Kingston, and were from both academic and community practices.

### Patient and caregiver questionnaire

All but one participant received a prescription for epinephrine. Two participants received prescriptions but did not fill them. Epinephrine was most commonly prescribed by allergists (46%) and family physicians (20%). Twenty-eight percent of participants (the caregiver, patient or both) reported not always carrying epinephrine auto-injectors. Participants received instruction on how to use auto-injectors from allergists (75%), family physicians (20%) and/or pharmacists (13%). Thirty-three percent of responders were not shown how to use an auto-injector with a trainer, and 43% were not asked to demonstrate how to administer epinephrine.

When patients were asked what information they received at diagnosis, most participants reported being given information on treating an allergic reaction (91%), how to recognize an allergic reaction (84%) and how to avoid relevant allergens (83%). However, fewer were given formation on medical identification such as MedicAlert^® ^(50%) and only 21% were given information about support groups.

Survey participants did not feel very confident about when to give an auto-injector (57%), how to administer it correctly (59%) or how to avoid food allergens (35%). Reasons for this lack of confidence were stated as not having used an auto-injector in a real situation (44%), lack of clarity on when to administer (19%) and fear (23%). Some respondents did not specify what they were afraid of, whereas others reported fear of "performing" in an emergency and fear of side effects.

Some survey respondents expressed that information was missing from their clinic visits (27%) and that more information was needed during future visits (65%). Participants who wanted more from their visits expressed the desire for information on the prevention and cure of food allergy (25%), more clarity around diagnosis (20%), how to avoid allergens (15%), and more information on social and emotional support (11%).

### Interview of allergists

Qualitative interviews with allergists showed that they ranked management of acute allergic reactions and teaching allergen avoidance as their top educational priorities. Major challenges for allergists included helping patients understand when to use an auto-injector, and to help them overcome the fear and reluctance associated with its use. Suggested strategies included: giving patients auto-injector trainers to take home for additional practice and teaching, providing patients with videos on how to use auto-injectors, and practicing auto-injector use with a trainer device with families at every visit.

Other common findings from the qualitative interviews were the challenges of limited time and human resources in clinic to ensure optimal care. Allergists indicated that nurse educators though potentially a great asset, were not really feasible due to lack of affordability. Alternative recommendations by allergists to improve efficiency of clinic visits were: using standardized check boxes in the chart as reminders to ensure all relevant topics are covered, incorporating electronic medical records into the practice, and providing links to reputable support groups who could help with patient teaching and psychosocial support.

Allergists also indicated that the support of families outside of clinic visits was a challenge. They suggested connecting patients to support groups, a telephone help line, and the provision of educational material for home use. Many also felt that improvement was also needed at the community level, such as high quality educational materials for teacher training, and first aid courses.

## Discussion

Our study highlights the experience and educational needs of 92 food allergic patients in Ontario as well as the challenges faced by 6 of their allergists. The majority of our data were collected from academic practices (67%) indicating improvements may need to occur in these settings. Though these same conclusions may apply to community clinics as well, these did not make up the bulk of our sample. We found three key areas in food allergy management that require improvement.

### Education and support of food allergic patients

Patients and caregivers indicated that both allergists and family physicians provided information about the recognition and treatment of allergic reactions, but both often failed to provide information on medical identification and/or support groups. This is important as evidence indicates that quality of life can be severely affected by food allergies in terms of restriction of social activities, increased fear and anxiety, lack of understanding by others, and feelings of isolation[5]. To improve support of patients, physicians need to better connect patients with reputable resources and support groups early on.

### Patients/caregivers knowledge and confidence regarding the use of epinephrine auto-injectors

Significant deficiencies were evident in the areas of auto-injector education. Our study showed that 33% of patients were not shown how to use an auto-injector with a trainer device, and 43% were not asked to demonstrate the use of an auto-injector even after 4 or 5 visits with an allergist. Most allergists, however, identified this area as a teaching priority. Furthermore, only about 30% of patients or caregivers felt very confident about when and how to give an auto-injector. This finding is consistent with another study of 101 food allergic families, which found that only 32% correctly demonstrated the use of the auto-injector using a trainer [8]. Based on these findings, we recommend that every physician visit should include practice with an auto-injector training device, and review of indications for its use. Since time in clinic is limited, it would be helpful to provide auto-injector trainers and multimedia material for patients to use at home and for teaching others.

### Changes needed at the community level

Findings of our study indicated that patients need to be supported beyond the clinic, perhaps through provision of standardized, accessible educational programs to train all caregivers, as well as primary care and specialist physicians. Previous studies have shown that knowledge and management of food allergy by primary care physicians and pediatricians are suboptimal. A 2000 study of 29 pediatricians at Mount Sinai Hospital in New York City found that only 18% were able to correctly demonstrate how to use an auto-injector [8]. Similarly, a 2008 study of 82 primary care and specialist physicians found that 23% were unable to correctly demonstrate use of the Epipen^®^, and 30% answered incorrectly on a question addressing the clinical presentation of anaphylaxis [9]. A study on the development of food allergy educational resources for primary care physicians, found that physicians preferred small group, on-site training [9]. Similar to first aid programs, we believe that a training program for patients should ideally be small group based, and moderated by trained personnel who can lead participants through scenarios. Most caregivers in our survey did not feel very confident using an auto-injector because they had never used one in a real situation, were fearful of the unknown, and/or were not clear on indications for its use. Similar to training physicians on how to deal with cardiac arrests, going through simulations of anaphylactic reactions may help participants gain practical knowledge and improve their self confidence.

### Strengths and limitations

To our knowledge, this is the first Canadian study to assess the experiences and educational needs of food allergic patients and caregivers, and the challenges faced by their treating allergists. The study also successfully piloted a questionnaire that measured patient and caregiver educational experiences and needs, which may be used in future studies to assess the impact of intervention programs, and used as a self evaluation tool by allergists.

There were limitations in our sampling. Our questionnaire and interview samples were limited by the small number of participants. Recall bias is another limitation. We attempted to reduce recall bias by giving participants the choice of indicating that they did not recall what information they received. Seven participants choose this option. However, it is possible that participants may not accurately remember all information requested, as they were on average at their 4.6 visit with the allergist when they completed the questionnaire. For example, it is possible that when participants were asked what information they received at their diagnosis, they reported less information than they actually received. Furthermore, despite our efforts to recruit participants from a wide variety of practice settings, the majority of respondents were recruited from academic allergy clinics. Two of the 6 allergists were from a community practice, and only 15 people from their practices participated in the study. As a result, our findings may not be generalizable to community allergy practices. Broadening our sampling to these populations is planned for future studies. Lastly, our study did not inquire about the existing teaching resources at academic and community offices. To the best of our knowledge, there is no published study evaluating teaching resources at allergists' offices. Future study would be needed to clarify what optimal resources would be in this context.

## Conclusions

Current experiences of families and their allergists indicate that teaching around the use of the epinephrine auto-injector needs to be a priority, ideally taught through small group scenario based programs. Physicians need to improve in their provision of information on medical identification and support groups.

## Competing interests

The authors declare that they have no competing interests.

## Authors' contributions

YSX and SW conceived the study, designed the study and wrote the paper. MK participated in its design and writing. LC and SBW helped to edit the manuscript. KS helped to collect the data for analysis. All authors read and approved the final manuscript.

## Supplementary Material

Additional file 1 **Caregiver questionnaire**. Click here for file
