# Supplementary material for: Food allergy management from the perspective of patients or caregivers, and allergists: a qualitative study
Source: Allergy Asthma Clin Immunol. 2010 Nov 30;6(1):30. doi: 10.1186/1710-1492-6-30 (PMC3002337; doi:10.1186/1710-1492-6-30)
Supplement: Additional file 1 — Caregiver questionnaire. [file 1710-1492-6-30-S1.DOC]

Allergist initials:

Date:

Time:

**Food Allergy Questionnaire**

**(For Substitute Decision Maker)**

**Background Information**

| 1. **What is your relationship to the patient?** |
| --- |
| 1. **Who regularly attends clinic appointments with the patient?** |
| 1. **How many times has your child been to this clinic?** |
| 1. **How long did you wait to get this appointment?** |
| 1. **What is the reason for your visit today?** |
| 1. **What was the patient’s age when he/she had the first adverse reaction?** |
| 1. **How old is the person with the allergy now?** |

**8. The patient is allergic to** *(please check ALL that apply)*:

| - Milk | - Egg | - Peanut | - Other nuts | - Wheat | - Sesame |
| --- | --- | --- | --- | --- | --- |
| - Soy | - Seafood | - Other foods *(please specify)*: | | | |

**9. Who helped to diagnose the patient’s allergy *(please check ALL that apply)*:**

| - Family physician | - Allergist | - Emergency physician | - Naturopath |
| --- | --- | --- | --- |
| - General pediatrician | - Other *(please specify)*: | | |

**Allergy Teaching**

| **10. Were you given a prescription for an epinephrine auto-injector (e.g. EpiPen or Twinject)?** | | - Yes | - No | - Do not know |
| --- | --- | --- | --- | --- |
|  | If YES, who prescribed the epinephrine pen? | | | |
| **11. Did you fill the prescription for the epinephrine auto-injector?** | | - Yes | - No | - Does not apply |
|  | If NO, please explain why: | | | |
| **12. Who taught you how to use an epinephrine auto-injector?** | | - Family Physician | - Allergist | - Other   (*Please specify*)  __________ |
|  | Did they use a trainer (a trainer looks like the auto-injector but does not contain medication or needle)? | - Yes | - No | - Do not know |
|  | Did they ask you to show them how to use it? | - Yes | - No | - Do not know |
| ***13. What information did you get when the allergy was diagnosed?***(please check all that apply)   |  | How to recognize an allergic reaction | | --- | --- | |  | How to avoid food allergens | |  | How to treat an allergic reaction | |  | MedicAlert identification | |  | Patient support groups | |  |  | |  | I do not remember what information I got | |  | I did not receive any information | | | | | |

**Managing Allergy**

| **14. Are you thinking about making changes to improve your child’s allergy management?**  *(please check ONE)*   - I am not thinking about making changes - I am thinking about making some changes - I am becoming determined to make changes - I am actively making changes - Other *(please specific):* |
| --- |
| **15. How confident do you feel about managing your child’s allergies?**  *(Please rate your confidence on a scale of 1 to 5)*  **Not confident Very confident**   | Knowing how to avoid food allergens | 1 | 2 | 3 | 4 | 5 | | --- | --- | --- | --- | --- | --- | | Knowing when to give the auto-injector | 1 | 2 | 3 | 4 | 5 | | Giving the auto-injector correctly | 1 | 2 | 3 | 4 | 5 | |
| **16. If you did not choose 5 (very confident) for any of the above, please explain why:** |
| ***17. Who carries the auto-injector?*** (please check all that apply)   | The person with the allergy carries the injector: | - Always | - Sometimes | - Never | | --- | --- | --- | --- | | A caregiver carries the injector (caregiver can be family member, babysitter, nanny, teacher, coach): | - Always | - Sometimes | - Never | |
| **18. How many times have you used the auto-injector?** |
| 1. **Where on the body would you give the auto-injector?** |
| 1. **Would you call 911 after giving the auto-injector and the allergic symptoms went away?** |
| 1. **Have you taught others (e.g. relatives, babysitters, teachers) how to use an auto-injector?** |
| 1. **How many times did your child accidentally eat an allergic food, after the diagnosis of allergy?** |

**Impact on Life**

| 1. **How has the allergy affected your child’s social / home life?** |
| --- |
| 1. **How has the allergy affected your child’s work / school life?** |

**Learning needs**

| 1. **What information was missing from your clinic visit(s)?** |
| --- |
| 1. **What else would you like to learn more about during future visits?** |

| 1. **Would you like to be contacted about participating in a study on improving teaching for patients with food allergies?** | | - Yes | - No thanks |
| --- | --- | --- | --- |
|  | **If your answer is YES,** please write your contact information on the next page, which will be collected separately so your answers above will remain anonymous. | | |

**Please tear off this page and return it to the research team. This will help us keep your information above anonymous.**

I wish to hear more about a study on improving teaching for patients with food allergies. My contact information is:

| **Name:** |  | |
| --- | --- | --- |
| **Telephone number:** |  | |
| **Email:** |  | |
| **I prefer to be contacted by:** | - **Phone** | - **Email** |
